# Entrustable Professional Activities for Chinese Standardized Residency Training in Pediatric Intensive Care Medicine

**DOI:** 10.3389/fped.2022.919481

**Published:** 2022-07-04

**Authors:** Zhang Yun, Liu Jing, Chen Junfei, Zhang Wenjing, Wu Jinxiang, Yue Tong, Zhang Aijun

**Affiliations:** ^1^Department of Pediatrics, Qilu Hospital of Shandong University, Jinan, China; ^2^Department of Biostatistics, School of Public Health, Cheeloo College of Medicine, Shandong University, Jinan, China; ^3^Department of Pediatric Surgery, Qilu Hospital of Shandong University, Jinan, China; ^4^Department of Obstetrics and Gynecology, Qilu Hospital of Shandong University, Jinan, China; ^5^Department of Pulmonary and Critical Care Medicine, Qilu Hospital of Shandong University, Jinan, China; ^6^Medical Training Office, Qilu Hospital of Shandong University, Jinan, China

**Keywords:** entrustable professional activities (EPA), pediatric intensive care medicine, standardized residency training (SRT), Chinese, assessment and education

## Abstract

**Background:**

Entrustable professional activities (EPAs) were first introduced by Olle ten Cate in 2005. Since then, hundreds of applications in medical research have been reported worldwide. However, few studies discuss the use of EPAs for residency training in pediatric intensive care medicine. We conducted a pilot study of EPA for pediatric intensive care medicine to evaluate the use of EPAs in this subspecialty.

**Materials and Methods:**

A cross-sectional study was implemented in pediatric intensive care medicine standardized residency training at the Qilu Hospital of Shandong University. An electronic survey assessing EPA performance using eight scales composed of 15 categories were distributed among residents and directors.

**Results:**

A total of 217 director-assessment and 44 residents’ self-assessment questionnaires were collected, both demonstrating a rising trend in scores across postgraduate years. There were significant differences in PGY1-vs.-PGY2 and PGY1-vs.-PGY3 director-assessment scores, while there were no differences in PGY2-vs.-PGY3 scores. PGY had a significant effect on the score of each EPA, while position significantly affected the scores of all EPAs except for EPA1 (Admit a patient) and EPA2 (Select and interpret auxiliary examinations). Gender only significantly affected the scores of EPA6 (Report a case), EPA12 (Perform health education), and EPA13 (Inform bad news).

**Conclusion:**

This study indicates that EPA assessments have a certain discriminating capability among different PGYs in Chinese standardized residency training in pediatric intensive care medicine. Postgraduate year, gender, and resident position affected EPA scores to a certain extent. Given the inconsistency between resident-assessed and director-assessed scores, an improved feedback program is needed in the future.

## Introduction

Entrustable professional activities (EPAs) were formally conceptualized in 2005 by Olle ten Cate, who defined EPAs as “units of professional practice, defined as tasks or responsibilities to be entrusted to the unsupervised execution by a trainee once he or she has attained sufficient specific competence” (1). The focus of competency-based medical education (CBME) in the recent years has been on achieving EPAs, which at present are likely the most widespread approach to CBME worldwide (2–7). It is indispensable that supervising consultants need a valid and reliable assessment tool of a learner’s performance, helping both realize the learner’s real abilities and improve in time. EPAs seem to be the optimal choice. Despite the seemingly universal affinity for EPAs, there is limited empirical evidence for their use in trainee assessment and a paucity of feedback about their clinical implementation.

It has become clear over the past decade that various EPA phenotypes exist worldwide (8–10). These phenotypes may vary based on how they define a stage of training or a profession. These differences may also reflect regulatory oversight in different regions, with some having a single regulatory body that facilitates alignment across the continuum and others having different regulatory bodies overseeing different phases of training and practice (7, 11–15). 35 evaluated a formative assessment system based on EPAs in pediatric residency training at the Peking University First Hospital, proposing an EPA system to assess postgraduate medical education (PGME) that was made up of 15 EPA categories on eight scales (16, 17). This highlighted the complementary advantage of EPAs that could be integrated with an ongoing CBME formative assessment program, including mini-clinical-evaluation exercises (Mini-CEX), direct observation of procedural skills (DOPSs), subjective-objective-assessment-plan (SOAP), and 360-degree assessment. Hence, in the last year, we began to push forward an EPA assessment program at the Qilu Hospital in Shandong University based on the CBME system for standardized residency training at the Peking University First Hospital.

Entrustable professional activities have been developed and published for a variety of pediatric subspecialties (18–23), as an emerging and practical tool for assessing trainees’ clinical competencies. Hennus et al. (19) reported a nationally modified Delphi study on developing a set of EPAs for Dutch pediatric intensive care medicine fellows. But few works discuss EPAs for residency training in pediatric intensive care medicine. To preliminarily explore the effectiveness of EPAs and deficiencies in residency training, we, therefore, performed a pilot study of 44 residents within the Chinese standardized residency training program in the pediatric intensive care medicine department at the Qilu Hospital of Shandong University and solicited both resident self-assessment and director-assessment of this training model.

## Materials and Methods

### Setting

Like many other Chinese standardized training residency programs, Qilu Hospital of Shandong University has a CBME evaluation course that spans the resident’s training after graduation from medical school and includes Mini-CEX, DOPS, SOAP, and 360-degree assessment. According to the national guidelines for standardized residency training, every pediatric resident is supposed to rotate through Pediatric Hematology, Urology, Neurology, Respiratory, Neonatology, Angiocardiopathy, Gastroenterology, Outpatient and Emergency, Infectious Diseases, and Child Healthcare subspecialties for at least 3 months within a 3-year training phase. The departmental rotation examination is administered at the end of each subspecialty rotation phase and is composed of all of the aforementioned skill tests and formative assessments. Directors in charge of pediatric intensive care medicine were pediatric intensive care unit physicians well-trained by the national or provincial director course for Chinese standardized training residency program, who obtained qualification certifications from the Chinese Health Commission or Shandong Provincial Health Commission.

### Sample

This study enrolled 44 residents who were trained in pediatric intensive care medicine as part of a standardized residency training program from January 2021 to February 2022 at the Qilu Hospital of Shandong University. In total, seven directors in charge of pediatric intensive care medicine over the same time period were also recruited for this study. All the enrolled residents were categorized into postgraduate year 1 (PGY1) to PGY3 according to their seniority. The study was approved by the Qilu Hospital of Shandong University Institutional Review Board.

### Procedure

Entrustable professional activity resident self-assessments and director-assessments were used at the end of the pediatric intensive care department rotation to evaluate resident performance and competency from both points of view. An electronic questionnaire composed of EPAs with 15 categories on eight scales was administered to solicit both resident self-assessment and director assessment in addition to the ongoing evaluation program (Mini-CEX, DOPS, SOAP, and 360-degree assessment). The director assessments of each resident were performed by several directors, whereas, the self-assessment of each resident was performed by the resident his/herself. Each questionnaire included general information (director name, resident name, resident gender, seniority, and position such as professional master, entrusted training residents from junior hospitals, residents of permanent staff at the Qilu Hospital of Shandong University, and social training residents) and EPA evaluation. The 15 categories of the EPA evaluation were established using the guidelines of the Peking University First Hospital (Table 1; 6). Based on the previous literature (16), each EPA was set using eight scales (Table 2). All the EPA assessments were performed until participating residents or directors were well-informed about all of the details of this questionnaire. All the questionnaires were conducted electronically *via* mobile software. Multiple reminders and phone follow-ups by data collection staff were set to ensure all required responses were collected in time. Each enrolled questionnaire result indicates that all of the included questions were completed.

**TABLE 1:** Entrustable professional activities (EPAs) categories.

Number	Category
1	Admit a patient
2	Select and interpret auxiliary examinations
3	Diagnose and make the differential diagnosis
4	Make therapeutic decision
5	Compose medical documents
6	Report a case
7	Recognize and manage general clinical conditions
8	Recognize and manage emergent and critical conditions
9	Transfer and hand over a patient
10	Perform informed consent
11	Perform basic operation
12	Perform health education
13	Inform bad news
14	Perform clinical education
15	Manage public health events

**TABLE 2:** Eight entrustable levels of each entrustable professional activity (EPA).

Scale	Details
1	Cannot perform certain professional activities as a resident under the direct supervision of a superior physician
2	Perform certain professional activities with a superior physician together
3	Perform certain professional activities under the supervision and guidance of a superior physician
4	Perform certain professional activities without the presence of a superior physician; when help is needed, need the presence of a superior physician to recheck all performances.
5	Perform certain professional activities without the presence of a superior physician; when help is needed, need the presence of a superior physician to recheck important performances.
6	Perform certain professional activities without the presence of the superior physician; when help is needed, need the guidance and recheck of superior physician over the phone.
7	Perform certain professional activities without the need for supervision and guidance from a superior physician.
8	Can provide supervision and guidance for others in certain professional activities.

### Statistical Analysis

All the questionnaires were administered using the Wenjuanwang APP 2.7.0 (Zhongyan Network Technology Co., Ltd., Shanghai, China). Data collection was performed using Excel (Microsoft, Redwood, WA, United States), and statistical analysis and figure creation were performed using SPSS 23.0.0 (IBM, Armonk, NY, United States). Comparisons between self-assessments and director-assessments for every EPA across different PGYs were statistically analyzed using the Kruskal–Wallis test. A two-sided *p* < 0.05 was considered statistically significant. Comparisons between self-assessments and director-assessments for every EPA between every two PGY levels were statistically analyzed using the Mann–Whitney U test, with significance defined as a corrected *p*-value of 0.017 using the Bonferroni correction for three times the Mann–Whitney U test for the same EPA. The effect analysis of PGY, gender, and position on the EPA scores of director assessments was analyzed using the generalized estimated equation (GEE), with *p* < 0.05 considered statistically significant.

## Results

### General Information

**FIGURE 1:**
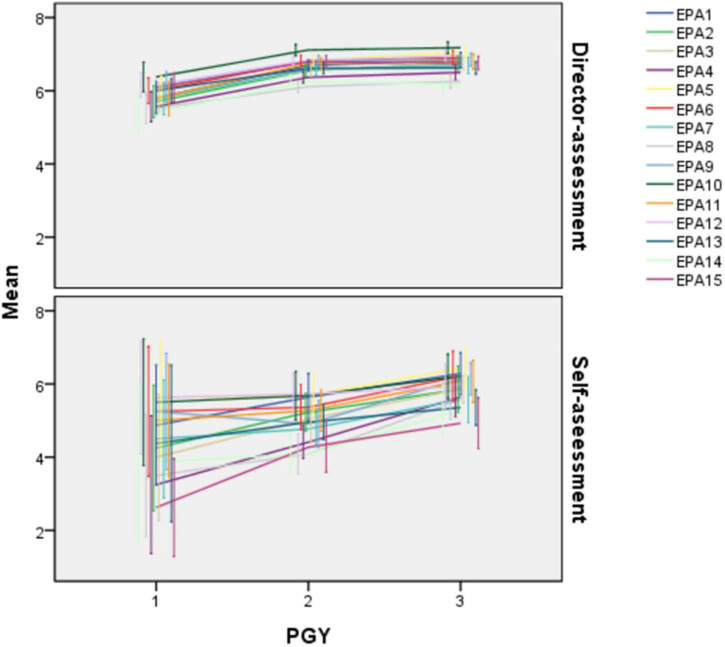
Line graph of scores of director-assessment and self-assessment in each entrustable professional activity (EPA). Each point stands for the mean of scores of a certain subgroup, with bars standing for the 95% CI of the mean of each subgroup.

This study recruited 44 residents (Table 3) and seven directors. The collected results included 44 resident self-assessment questionnaires and 217 director-assessment questionnaires, with a 100% response rate. The number of director-assessment and self-assessment questionnaire results are listed in Table 3. A line graph was created to show the trend in director-assessment and self-assessment EPA scores over progressive PGY levels (Figure 1). A slowly rising trend in director-assessment scores across all the EPA by PGY year was noted, while self-assessment scores showed a non-distinctive trend across different PGYs.

**TABLE 3:** Characteristics of residents.

Characteristics	PGY1	PGY2	PGY3	*p*-value
Number of residents, *n* (%)	8 (18.2%)	22 (50.0%)	14 (31.2%)	−
Male, *n* (%)	2 (25.0%)	4 (18.2%)	2 (14.3%)	0.82
Number of director-assessments, mean ± SD	4.6 ± 1.5	4.7 ± 1.2	5.1 ± 0.5	0.53

### Comparison of Director-Assessment Scores Across Different Postgraduate Years

Director-assessment EPA scores are listed in Table 4. There were significant differences between the EPA scores across different PGYs. The higher the PGY year that the residents were in, the higher the scores that they got. When univariate PGY years were compared, there were significant differences between PGY1 and PGY2 and between PGY1 and PGY3 (*p* < 0.017), whereas there were no obvious differences between PGY2 and PGY3 in any EPA category.

**TABLE 4:** Scores of director-assessment in different postgraduate years (PGYs).

EPAs	PGY1	PGY2	PGY3	Chi-square*	*P*-value
EPA1	5.8 ± 1.3**	6.7 ± 0.8	6.8 ± 0.9***	21.293	0.000
EPA2	5.7 ± 1.3**	6.6 ± 0.8	6.8 ± 0.8***	22.256	0.000
EPA3	5.8 ± 1.2**	6.6 ± 0.8	6.7 ± 0.7***	18.764	0.000
EPA4	5.6 ± 1.2**	6.4 ± 0.8	6.5 ± 0.9***	18.428	0.000
EPA5	6.0 ± 1.1**	6.8 ± 0.7	7.1 ± 0.7***	26.320	0.000
EPA6	6.0 ± 1.1**	6.8 ± 0.7	6.9 ± 0.8***	22.902	0.000
EPA7	5.8 ± 1.3**	6.6 ± 0.8	6.7 ± 1.0***	15.798	0.000
EPA8	5.5 ± 1.2**	6.1 ± 0.9	6.3 ± 0.8***	13.614	0.001
EPA9	6.1 ± 1.2**	6.8 ± 0.8	6.9 ± 0.8***	12.933	0.002
EPA10	6.4 ± 1.2**	7.1 ± 0.8	7.2 ± 0.7***	13.510	0.001
EPA11	5.8 ± 1.4**	6.7 ± 0.8	6.8 ± 0.9***	17.453	0.000
EPA12	6.2 ± 1.0**	6.8 ± 0.7	7.0 ± 0.6***	23.717	0.000
EPA13	6.0 ± 1.0**	6.6 ± 0.8	6.6 ± 0.7***	13.989	0.001
EPA14	5.4 ± 1.5**	6.3 ± 0.9	6.2 ± 0.9***	8.881	0.012
EPA15	6.1 ± 1.2**	6.8 ± 0.9	6.8 ± 0.8***	11.238	0.004

*Kruskal–Wallis test. **Mann–Whitney U test revealed a significant difference between PGY1 and PGY2. ***Mann–Whitney U test revealed a significant difference between PGY1 and PGY3.

### Effect Analysis of Postgraduate Year, Gender, and Position on Director-Assessment Scores

Given that resident PGY, gender, and position all could affect director EPA scores (Table 5), a GEE model analysis was performed to analyze the effect of these factors on EPAs score (Table 6). PGY had a significant effect on all EPA scores (*p* < 0.05), whereas, resident position significantly affected every EPA score except for EPA1 (*p* = 0.714, >0.05) and EPA2 (*p* = 0.076, >0.05). Resident gender only significantly affected EPA6 (*p* = 0.002, <0.05), EPA12 (*p* = 0.010, <0.05), and EPA13 (*p* = 0.018, <0.05) (Table 6).

**TABLE 5:** Categorical variable information of director-assessment questionnaires.

Factor		*N*	Percent
PGY	PGY1	37	17.1%
	PGY2	106	65.9%
	PGY3	74	34.1%
Gender	Female	177	81.6%
	Male	40	18.4%
Position	Professional master	118	54.4%
	Entrusted training residents	82	37.8%
	Permanent staff	8	3.7%
	Social training residents	9	4.1%

**TABLE 6:** Generalized estimated equation analysis of director-assessment questionnaires.

EPAs	Factor	Tests of model effects		Parameter	B	95% Wald confidence interval			Hypothesis test
						
		Ward Chi-square	*P*-value			Lower	Upper	Wald Chi-square	*P*-value
EPA1	PGY	11.827	0.003	PGY1	0^a^				
				PGY2	0.794	0.270	1.318	8.816	0.003
				PGY3	0.988	0.425	1.551	11.820	0.001
	Gender	1.814	0.178	Male	0^a^				
				Female	–0.278	–0.684	0.127	1.814	0.178
	Position	1.363	0.714	Professional master	0^a^				
				Entrusted training residents	0.168	–0.131	0.460	1.194	0.275
				Permanent staff	0.091	–0.164	0.346	0.486	0.484
				Social training residents	0.129	–0.199	0.457	0.594	0.441
EPA2	PGY	18.158	0.000	PGY1	0^a^				
				PGY2	0.712	0.214	1.211	7.855	0.005
				PGY3	0.983	0.485	1.481	14.987	0.000
	Gender	2.395	0.122	Male	0^a^				
				Female	–0.274	–0.621	0.073	2.395	0.122
	Position	6.870	0.076	Professional master	0^a^				
				Entrusted training residents	0.237	0.021	0.609	4.409	0.036
				Permanent staff	0.154	–0.054	0.362	2.115	0.146
				Social training residents	0.315	0.019	0.455	4.557	0.033
EPA3	PGY	20.128	0.000	PGY1	0^a^				
				PGY2	0.541	0.143	0.939	7.092	0.008
				PGY3	0.812	0.419	1.206	16.392	0.000
	Gender	2.637	0.104	Male	0^a^				
				Female	–0.247	–0.545	0.051	2.637	0.104
	Position	13.083	0.004	Professional master	0^a^				
				Entrusted training residents	0.350	0.149	0.551	11.628	0.001
				Permanent staff	0.334	0.130	0.538	10.325	0.001
				Social training residents	0.263	–0.051	0.578	2.689	0.101
EPA4	PGY	15.347	0.000	PGY1	0^a^				
				PGY2	0.695	0.238	1.152	8.887	0.003
				PGY3	0.891	0.442	1.339	15.135	0.000
	Gender	1.219	0.270	Male	0^a^				
				Female	–0.211	–0.586	0.164	1.219	0.270
	Position	59.347	0.000	Professional master	0^a^				
				Entrusted training residents	0.144	–0.127	0.415	1.088	0.003
				Permanent staff	0.042	–0.234	0.318	0.090	0.796
				Social training residents	0.446	0.151	0.741	8.788	0.003
EPA5	PGY	17.812	0.000	PGY1	0^a^				
				PGY2	0.681	0.263	1.100	10.167	0.001
				PGY3	0.981	0.514	1.448	16.947	0.000
	Gender	2.899	0.089	Male	0^a^				
				Female	–0.261	–0.561	0.039	2.899	0.089
	Position	192.621	0.000	Professional master	0^a^				
				Entrusted training residents	0.180	–0.040	0.400	2.579	0.108
				Permanent staff	0.360	0.188	0.531	16.929	0.000
				Social training residents	0.137	–0.038	0.311	2.356	0.125
EPA6	PGY	30.904	0.000	PGY1	0^a^				
				PGY2	0.723	0.423	1.023	22.370	0.000
				PGY3	0.884	0.567	1.200	29.940	0.000
	Gender	9.606	0.002	Male	0^a^				
				Female	–0.394	–0.644	-0.145	9.606	0.002
	Position	19.319	0.000	Professional master	0^a^				
				Entrusted training residents	0.146	0.149	0.551	11.628	0.199
				Permanent staff	0.381	0.185	0.577	14.525	0.000
				Social training residents	0.279	0.047	0.511	5.568	0.018
EPA7	PGY	18.023	0.000	PGY1	0^a^				
				PGY2	0.668	0.306	1.029	13.120	0.000
				PGY3	0.802	0.431	1.173	17.928	0.000
	Gender	1.858	0.173	Male	0^a^				
				Female	–0.224	–0.546	0.098	1.858	0.173
	Position	10.106	0.018	Professional master	0^a^				
				Entrusted training residents	0.233	0.038	0.428	5.466	0.019
				Permanent staff	0.232	0.079	0.385	8.824	0.003
				Social training residents	0.132	–0.138	0.403	0.918	0.338
EPA8	PGY	10.960	0.004	PGY1	0^a^				
				PGY2	0.449	0.067	0.830	5.310	0.021
				PGY3	0.640	0.256	1.023	10.683	0.001
	Gender	2.118	0.146	Male	0^a^				
				Female	–0.231	–0.543	0.080	2.118	0.146
	Position	410.269	0.000	Professional master	0^a^				
				Entrusted training residents	0.288	0.037	0.539	5.056	0.025
				Permanent staff	0.234	–0.002	0.470	3.781	0.052
				Social training residents	0.039	–0.198	0.276	0.103	0.748
EPA9	PGY	13.064	0.001	PGY1	0^a^				
				PGY2	0.536	0.202	0.869	9.893	0.002
				PGY3	0.625	0.284	0.966	12.909	0.000
	Gender	1.523	0.217	Male	0^a^				
				Female	–0.190	–0.492	0.112	1.523	0.217
	Position	13.392	0.004	Professional master	0^a^				
				Entrusted training residents	0.350	0.113	0.550	8.833	0.003
				Permanent staff	0.027	–0.149	0.202	0.089	0.765
				Social training residents	0.178	–0.206	0.563	0.828	0.363
EPA10	PGY	12.457	0.002	PGY1	0^a^				
				PGY2	0.526	0.146	0.907	7.363	0.007
				PGY3	0.672	0.295	1.050	12.181	0.000
	Gender	3.703	0.054	Male	0^a^				
				Female	–0.299	–0.604	0.006	3.703	0.054
	Position	12.114	0.002	Professional master	0^a^				
				Entrusted training residents	0.370	0.149	0.551	11.628	0.001
				Permanent staff	0.424	0.221	0.626	16.861	0.000
				Social training residents	0.168	–0.034	0.370	2.645	0.104
EPA11	PGY	17.518	0.000	PGY1	0^a^				
				PGY2	0.787	0.384	1.191	14.629	0.000
				PGY3	0.900	0.478	1.322	17.489	0.000
	Gender	0.922	0.337	Male	0^a^				
				Female	–0.172	–0.523	0.179	0.922	0.337
	Position	11.541	0.009	Professional master	0^a^				
				Entrusted training residents	0.277	0.078	0.476	7.466	0.006
				Permanent staff	0.250	0.099	0.400	10.608	0.001
				Social training residents	0.242	–0.130	0.615	1.624	0.203
EPA12	PGY	18.068	0.000	PGY1	0^a^				
				PGY2	0.474	0.141	0.807	7.797	0.005
				PGY3	0.700	0.363	1.037	16.602	0.000
	Gender	6.576	0.010	Male	0^a^				
				Female	–0.346	–0.610	-0.082	6.576	0.010
	Position	487.267	0.000	Professional master	0^a^				
				Entrusted training residents	0.318	0.124	0.513	10.283	0.001
				Permanent staff	0.698	0.519	0.876	58.793	0.000
				Social training residents	–0.014	–0.205	0.177	0.020	0.888
EPA13	PGY	10.742	0.005	PGY1	0^a^				
				PGY2	0.428	0.089	0.768	6.118	0.013
				PGY3	0.555	0.223	0.888	10.707	0.001
	Gender	5.631	0.018	Male	0^a^				
				Female	–0.463	–0.845	-0.081	5.631	0.018
	Position	102.285	0.000	Professional master	0^a^				
				Entrusted training residents	0.277	0.149	0.551	11.628	0.020
				Permanent staff	0.809	0.130	0.538	10.325	0.000
				Social training residents	0.128	–0.179	0.436	0.669	0.414
EPA14	PGY	11.813	0.003	PGY1	0^a^				
				PGY2	0.753	0.318	1.188	11.516	0.001
				PGY3	0.693	0.271	1.116	10.337	0.001
	Gender	0.486	0.486	Male	0^a^				
				Female	–0.146	–0.556	0.264	0.486	0.486
	Position	26.481	0.000	Professional master	0^a^				
				Entrusted training residents	0.136	–0.618	0.429	0.126	0.278
				Permanent staff	0.431	0.227	0.634	17.161	0.000
				Social training residents	–0.095	–0.051	0.578	2.689	0.723
EPA15	PGY	13.682	0.001	PGY1	0^a^				
				PGY2	0.695	0.327	1.063	13.679	0.008
				PGY3	0.634	0.252	1.015	10.612	0.000
	Gender	1.099	0.295	Male	0^a^				
				Female	–0.191	–0.548	0.166	1.099	0.295
	Position	30.171	0.000	Professional master	0^a^				
				Entrusted training residents	0.152	0.149	0.551	11.628	0.146
				Permanent staff	–0.235	–0.377	-0.093	10.494	0.001
				Social training residents	–0.311	–0.462	-0.161	16.398	0.000

*^a^ Set to zero because this parameter is redundant.*

The scores of all 15 EPA categories rose as PGY grew except for EPA14 (perform clinical education, set PGY1 as zero; PGY2: B = 0.753, *p* = 0.001, <0.05, PGY3: B = 0.693, *p* = 0.001, <0.05) and EPA15 (Manage public health events, PGY2: B = 0.695, *p* = 0.000, <0.05, PGY3: B = 0.634, *p* = 0.001, <0.05), with higher mean scores for PGY2s than PGY3s and the lowest mean score at PGY1. The mean scores of male residents in EPA6 (Report a case, set male as zero; female: B = −0.394, *p* = 0.002, <0.05), EPA12 (Perform health education, female: B = −0.346, *p* = 0.010, <0.05), and EPA13 (Inform bad news, female: B = −0.463, *p* = 0.018, <0.05) were higher than those of females. Entrusted training residents got the highest scores in EPA3 (Diagnose and make differential diagnosis, set professional master as zero; B = 0.350, *p* = 0.001, <0.05), EPA7 (Recognize and manage general clinical conditions, B = 0.233, *p* = 0.019, <0.05), EPA8 (Recognize and manage emergent and critical conditions, B = 0.288, *p* = 0.025, <0.05), EPA9 (Transfer and hand over a patient, B = 0.332, *p* = 0.003, <0.05), EPA11(Perform basic operation, B = 0.277, *p* = 0.006, <0.05), while permanent staff ranked as the top subgroup in EPA5 (Compose medical documents, set professional master as zero; B = 0.360, *p* = 0.000, <0.05), EPA6 (Report a case, B = 0.381, *p* = 0.000, <0.05), EPA10 (Perform informed consent, B = 0.424, *p* = 0.000, <0.05), EPA12 (Perform health education, B = 0.698 *p* = 0.000, <0.05), EPA13 (Inform bad news, B = 0.809, *p* = 0.000, <0.05), and EPA14 (Perform clinical education, B = 0.431, *p* = 0.000, <0.05). Social training residents were the best subgroup in EPA2 (Select and interpret auxiliary examinations, set professional master as zero; B = 0.315, *p* = 0.036, <0.05) and EPA4 (Make therapeutic decision, B = 0.446, *p* = 0.003, <0.05) while professional masters performed best in EPA15 (Manage public health events, *p* < 0.05).

### Comparison of Self-Assessment Scales Across Different Postgraduate Years

Self-assessment EPA scores are listed in Table 7. There were significant differences only within EPA2 (Select and interpret auxiliary examinations), EPA3 (Diagnose and make the differential diagnosis), EPA4 (Make therapeutic decision), EPA8 (Recognize and manage emergent and critical conditions), EPA9 (Transfer and hand over a patient), EPA14 (Perform clinical education), and EPA15 (Manage public health events) across the different PGY years, with higher level PGY residents scoring better. There were no obvious differences in the other EPAs across different PGYs. As for the comparisons between the two PGYs, there were significant differences in EPA15 (Manage public health events) scores between PGY1 and PGY2 (*p* < 0.017), and in EPA3 (Diagnose and make the differential diagnosis), EPA4 (Make therapeutic decision), EPA8 (Recognize and manage emergent and critical conditions), EPA9 (Transfer and hand over a patient), and EPA14 (Perform clinical education) between PGY2 and PGY3 (*p* < 0.017). Significant differences in EPA3 (Diagnose and make the differential diagnosis), EPA4 (Make therapeutic decision), EPA8 (Recognize and manage emergent and critical conditions), and EPA15 (Manage public health events) were seen between PGY1 and PGY3 (*p* < 0.017).

**TABLE 7:** Scores of self-assessment in different postgraduate years (PGYs).

EPAs	PGY1	PGY2	PGY3	Chi-square*	*P*-value
EPA1	4.9 ± 2.0	5.6 ± 1.5	6.3 ± 1.0	4.474	0.107
EPA2	4.3 ± 2.1	5.2 ± 1.2	5.9 ± 1.1	6.449	0.040
EPA3	4.0 ± 2.1	5.0 ± 1.4**	5.9 ± 0.7***	9.610	0.008
EPA4	3.3 ± 2.3	4.4 ± 1.0**	5.6 ± 0.9***	14.779	0.001
EPA5	5.6 ± 1.8	5.7 ± 1.4	6.4 ± 0.9	2.367	0.306
EPA6	5.3 ± 2.1	5.4 ± 1.4	6.2 ± 1.2	2.727	0.256
EPA7	4.5 ± 2.0	4.8 ± 1.0	5.6 ± 1.1	5.764	0.056
EPA8	3.5 ± 2.0	4.1 ± 1.2**	5.6 ± 0.9***	13.582	0.001
EPA9	5.3 ± 1.9	4.9 ± 1.4**	6.1 ± 0.8	7.347	0.025
EPA10	5.5 ± 2.1	5.7 ± 1.5	6.2 ± 1.1	0.584	0.747
EPA11	5.0 ± 1.9	5.3 ± 1.3	6.1 ± 1.0	4.208	0.122
EPA12	5.6 ± 1.8	5.7 ± 1.3	5.9 ± 1.1	0.050	0.976
EPA13	4.4 ± 2.6	5.0 ± 1.1	5.4 ± 0.8	1.623	0.444
EPA14	3.9 ± 2.6	4.1 ± 1.2**	5.3 ± 1.2	6.614	0.037
EPA15	2.6 ± 1.6**	4.3 ± 1.5	4.9 ± 1.2***	10.802	0.005

*Kruskal–Wallis test.

**Mann–Whitney U test revealed a significant difference between PGY2 and PGY3.

***Mann–Whitney U test revealed a significant difference between PGY1 and PGY3.

### Comparison of Entrustable Professional Activities Scores of Self-Assessments Between Genders

**FIGURE 2:**
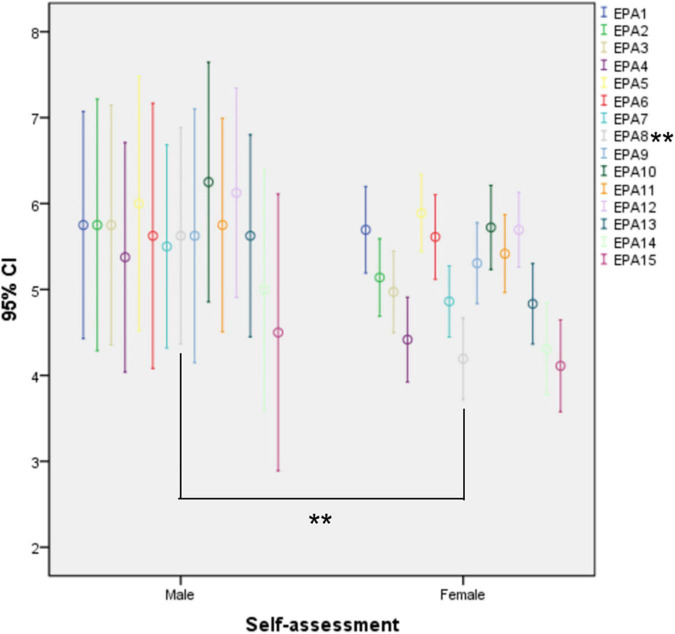
Error bar chart of self-assessment between genders. The edges of each bar stand for the 95% CI of scores in subgroups. **EPA8: *p* = 0.019, *p* < 0.05.

There was a significant difference in EPA8 (Recognize and manage emergent and critical conditions, *p* = 0.019, *p* < 0.05) between the self-assessment scores of male and female residents, with male residents self-scoring better than females (Figure 2).

### Comparisons Between Director and Self-Assessment Scores Across Entrustable Professional Activities Within the Same Postgraduate Year

**FIGURE 3:**
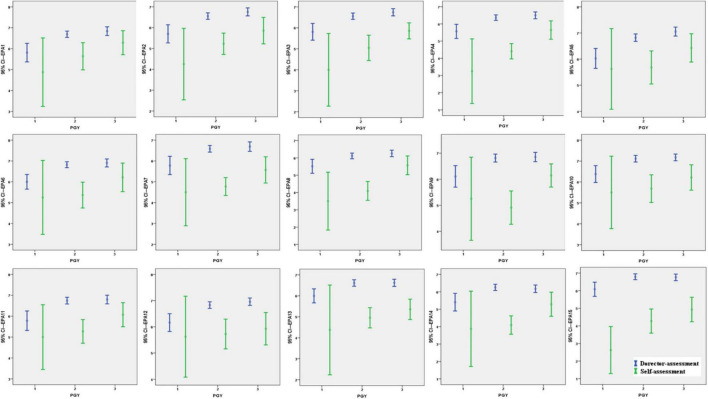
Comparison of director-assessment vs. self-assessment in entrustable professional activities (EPAs) within the same postgraduate year (PGY). The edges of each bar mean the 95% CI of scores of each subgroup in EPAs. The point of each bar in the middle stand for the mean of scores of a certain subgroup.

The director and self-assessment scores of PGY1s were mostly consistent except for EPA2 (Select and interpret auxiliary examinations, *p* = 0.31, *p* < 0.05), EPA3 (Diagnose and make a differential diagnosis, *p* = 0.12, *p* < 0.05), EPA4 (Make the therapeutic decision, *p* = 0.03, *p* < 0.05), EPA7 (Recognize and manage general clinical conditions, *p* = 0.39, *p* < 0.05), EPA8 (Recognize and manage emergent and critical conditions, *p* = 0.002, *p* < 0.05), and EPA15 (Manage public health events, *p* = 0.00, *p* < 0.05), where directors awarded higher scores. There were significant differences between the self-assessment and director-assessment scores for every EPA for PGY2s and PGY3s (PGY2: EPA1 *p* = 0.001, other EPAs *P* = 0.000; PGY3: EPA1 *p* = 0.036, EPA2 *P* = 0.003, EPA3 *P* = 0.000, EPA4 *P* = 0.002, EPA5 *P* = 0.012, EPA6 *P* = 0.034, EPA7 *P* = 0.001, EPA8 *P* = 0.008, EPA9 *P* = 0.002, EPA10 *P* = 0.001, EPA11 *P* = 0.014, EPA12 *P* = 0.000, EPA13 *P* = 0.000, EPA14 *P* = 0.009, EPA15 *P* = 0.000), with higher scores awarded by the director-assessment for each EPA (Figure 3).

## Discussion

Since their initial introduction by Olle ten Cate (1) in 2005, EPA have become an important part of CBME in undergraduate and postgraduate medical education settings (17, 19, 24). EPAs are designed to be real-life activities, and as such can be understood and applied more easily than prior concepts within CBME, such as milestones (25). An EPA combines the knowledge, skills, and attitudes necessary to perform a task, incorporating and synthesizing learning objectives into a meaningful unit. EPAs provide a framework to make judgments of trainee ability explicit, which is important at all stages of medical education (26). In their literature search, Kerth et al. (27) reported a notable shift from descriptions of EPA development processes toward aspects beyond development, such as implementation, feasibility, acceptance/perception, and assessment. Of note, there are few studies about EPAs in pediatric postgraduate education, of which most are from general pediatric residencies or other subspecialties, such as pediatric emergency medicine, pediatric cardiology, and neonatology. Furthermore, studies from Asia are scarce. This study focused on the implementation and feasibility of EPAs in Chinese standardized residency training in pediatric intensive care medicine.

Our study suggested that the director-assessment scores of residents in pediatric intensive care in every EPAs rose significantly over postgraduate training, with significant differences between PGY1 vs. PGY2 and PGY1 vs. PGY3 but not PGY2 vs. PGY3. These findings were nearly consistent with previous studies that utilized residency training programs (28) and fellows using American Board of Pediatrics subspecialty EPAs (29). However, with respect to self-assessment scores, only a segment of EPA scores were significantly different across PGYs and between individual PGY years. When an effect analysis on PGY, gender, and position on EPA scores was performed, EPA scores rose with PGY except for EPA14 and EPA15 while gender affected every EPA score significantly, with the male residents scoring higher. In contrast, residents in different positions scored better in different EPAs. The male self-assessment scores in “Recognize and managed emergent and critical conditions in pediatric intensive care” were significantly higher than female scores, while other EPAs were equivalent between genders. When self-assessment and director-assessment scores of PGY1s were compared, most of the director-assessed scores were significantly higher than those of the resident self-assessments. Furthermore, all of the director-assessment scores in every EPA category were significantly better than the self-assessment scores of both PGY2s and PGY3s.

This was a cross-sectional study in pediatric intensive care that evaluated the implementation and feasibility of EPAs in the formative assessment of the ongoing CBME for standardized residency training. The upward trend in director-assessed scores for each EPA over pediatric intensive care was significant. PGY1 residents are less capable of certain professional activities in pediatric intensive care than PGY2s and PGY3s, while there were no significant differences in any director-assessed score between PGY2 and PGY3 in pediatric intensive care. This obvious change in ability between the PGY1 and PGY2/PGY3 years may be due to the first years of training immediately after graduating, while there would be incremental development during the second or third year due to wide-ranging rotations across all the pediatric subspecialties instead of continuous training within one certain subspecialty. On the other hand, the insufficiency of professional activities during undergraduate education for trainees before standardized training was revealed based on the relatively lower scores of PGY1 residents. As stepped elevation is emphasized in the CBME program, residents are thought to develop their professional skills as their training time increases (30, 31). These areas include all EPAs, suggesting that the curriculum for training residents in these areas requires notable improvement, and directors and regulatory agencies should be encouraged to reinforce the idea up-grading professional skills between the PGY2 and PGY3 years (32, 33).

For most of these 15-category EPAs, there was a certain percentage of residents who were able to practice EPAs unsupervised by the end of 3 years of residency training. However, for the remaining group of unqualified residents to be able to practice those EPAs unsupervised by the end of their required training, educators and regulatory agencies would need to implement EPA-based assessments more broadly or efficiently in pediatric subspecialties, as suggested previously (34). If we expect residents to meet the standards for unsupervised practice after training in all 15 EPA categories, either training needs to be enhanced significantly in these areas or our expectations of what residents are required to achieve by the completion of their training need to be adjusted. Future studies should be performed to determine whether similar experiences have been reported in other specialties.

Given that PGY, gender, and position could affect EPA score, we used a GEE model to analyze our correlation analysis. EPA scores rose significantly across PGY years except for EPA14 (Perform clinical education) and EPA15 (Manage public health events), with the highest scores noted among PGY2s. This suggests a lack of stepwise training between the 2nd and 3rd year within this standardized training program. After the first postgraduate year of training, individual talents might be distinguishing each resident’s abilities. With respect to the gender gap, the scores for EPA6 (Report a case), EPA12 (Perform health education), and EPA13 (Inform bad news) were significantly higher among the male residents. After interviewing the enrolled directors, the potential advantages in the logical thinking and professional image credibility of male physicians in the daily workplace make this result understandable. There were four kinds of resident positions, which had an effect on EPA score differences. Professional masters had just graduated with their bachelor’s degrees from medical school while permanent staff mostly had doctoral degrees, which required a prolonged research period or more professional knowledge in some fields. Whereas, the entrusted training residents and social training residents were more experienced in clinical work and usually worked for a few years prior to attending standardized residency training, they generally had a lesser educational background. Different backgrounds led to different advantages in professional activities, which can allow us to reinforce the personalized training plan for residents in different positions to play to everyone’s strengths.

Resident self-assessment scores were inconsistent with the director’s perception. Residents believed that they had significantly developed only in EPA2 (Select and interpret auxiliary examinations), EPA3 (Diagnose and make the differential diagnosis), EPA4 (Make therapeutic decision), EPA8 (Recognize and manage emergent and critical conditions), EPA9 (Transfer and hand over a patient), EPA14 (Perform clinical education), and EPA15 (Manage public health events) over their 3 years of standardized training. There was a significant difference in self-assessment scores between genders only in EPA8 (Recognize and manage emergent and critical conditions), with males scoring higher. This might come from the male advantage in physical strength and adaptability to a heavy daily workload and the burden of the pediatric intensive care medicine rotation. Of note, there were a limited number of male residents enrolled in this study, which might lead to inconsistencies between director- and self-assessment scores. A further large cohort of residents is required to produce more reliable results.

The director-assessment scores were higher than the self-assessment scores of PGY1s in EPA2 (Select and interpret auxiliary examinations), EPA3 (Diagnose and make the differential diagnosis), EPA4 (Make therapeutic decision), EPA7 (Recognize and manage general clinical conditions), EPA8 (Recognize and manage emergent and critical conditions), and EPA15 (Manage public health events). Similar situations were found in the PGY2 and PGY3 years across all EPAs categories. This is likely due to the lack of self-confidence and self-recognition among the residents. It may also indicate the lack of efficient feedback from directors to residents, preventing the trainee’s understanding of how they performed and what they needed to improve. Further efficient feedback on EPAs is required.

Our study has several strengths. It reported the implementation and feasibility of EPAs in the Chinese standardized training of pediatric intensive care residents. It established obvious differences in EPA performance between lower PGY and higher PGY residents and provided a well-structured framework to guide residents in the development of the knowledge, skills, and attitudes necessary to perform a task while incorporating and synthesizing learning objectives. We analyzed the effects of PGY, gender, and resident position on EPAs scores, confirming that PGY and gender correlated with EPA scores while resident position had a limited impact. The incongruity between director-assessed and self-assessment scores indicates the need for an efficient feedback program.

There are also limitations to our study. First, the sample size is limited, leading to our inability to analyze the reliability and validity of EPA implementation in pediatric intensive care medicine training. This was limited by the capability of resident training at our hospital and the number of directors at our institution. The translation into clinical practice and how these skills affect the patient outcome remains to be determined. Second, this is a cross-sectional study that enrolled residents trained in pediatric intensive care medicine within the last year. There are no detailed outcomes related to clinical practice and patient outcomes measured. Since EPAs were newly integrated into the ongoing CBME program in China, we had limited experience with this. A longitudinal study may be validated, and a multicenter longitudinal study would be of great value.

In summary, this study indicates that EPA assessments had a certain discriminating capability between class years of Chinese standardized residency training in pediatric intensive care medicine, with scores rising with PGY year. Postgraduate year, gender, and resident position impacted EPA scores. Given the incongruities between resident-assessed and director-assessed scores, an improved feedback program is needed.

## Data Availability Statement

The raw data supporting the conclusions of this article will be made available by the authors, without undue reservation.

## Ethics Statement

The studies involving human participants were reviewed and approved by Qilu Hospital of Shandong University Institutional Review Board. The patients/participants provided their written informed consent to participate in this study.

## Author Contributions

ZY initiated the study, participated in the design and coordination, did the basic statistical analysis, and drafted the manuscript. LJ did the majority of the statistical analysis. ZA helped to initiate the study and edit the manuscript. CJ, ZW, WJ, and YT helped to collect the original data and did the statistical analysis. All authors read and approved the final manuscript.

## Conflict of Interest

The authors declare that the research was conducted in the absence of any commercial or financial relationships that could be construed as a potential conflict of interest.

## Publisher’s Note

All claims expressed in this article are solely those of the authors and do not necessarily represent those of their affiliated organizations, or those of the publisher, the editors and the reviewers. Any product that may be evaluated in this article, or claim that may be made by its manufacturer, is not guaranteed or endorsed by the publisher.
